# Examination of the Usefulness of Standby Therapy for Refractory Chylous Ascites After Multiple Lymphangiography Interventions

**DOI:** 10.7759/cureus.62735

**Published:** 2024-06-19

**Authors:** Yu Horibe, Mao Kunishi, Toshiyuki Kanno, Takashi Motohashi, Tsutomu Tabata

**Affiliations:** 1 Gynecology, Tokyo Women’s Medical University, Tokyo, JPN; 2 Obstetrics and Gynecology, Tokyo Women’s Medical University, Tokyo, JPN

**Keywords:** lymph node dissection, lymphangiography, gynecologic malignancy, standby therapy, chylous

## Abstract

Chylous ascites is infrequently observed following lymph node dissection in surgeries for gynecological malignancies. If symptoms develop, they can severely debilitate patients and increase the risk of infection, particularly those with a low performance status following the primary operation. Treatment of chylous ascites is often challenging and protracted, with no treatment currently guaranteeing a complete cure. This study explores the efficacy of standby therapy for refractory chylous ascites in a 46-year-old woman with gynecological malignancies who did not respond to multiple lymphangiographic interventions. Due to a suspicion of left ovarian cancer, she underwent surgery including lymph node dissection. On the following day, significant amounts of ascites were confirmed in the abdominal cavity. Despite performing lymphangiography twice, the chylous ascites persisted. During follow-up in the outpatient ward, on the 142nd post-surgery day, the ascites had spontaneously resolved. In cases like this, where symptoms are relatively mild and surgical intervention is not preferred due to complications or patient preference following lymphangiography, it may be beneficial to use standby therapy in combination with dietary management during outpatient follow-up. Such an approach could yield medium- to long-term improvements and should be considered. However, if further treatment is planned following the initial surgery, the patient’s long-term prognosis should be considered, and treatment should be administered promptly. Various methods exist for treating refractory chylous ascites, including expectant therapy, dietary management, percutaneous drainage, lymphangiography and embolization, and surgical lymphatic ligation. Tailoring individualized treatment plans for each patient and pursuing a multidisciplinary approach is advisable. Although initiating adjuvant chemotherapy may not be feasible, long-term standby therapy is beneficial, even if lymphangiography proves ineffective in the short term.

## Introduction

Chylous ascites is characterized by three primary mechanisms, namely, the exudation of lymph through the walls of congenital or acquired vessels into the peritoneal cavity, malignant infiltration of the lymphatic system causing leakage from the subserosal lymphatics, and direct injury (traumatic or iatrogenic) to the retroperitoneal lymphatics [1]. This condition is infrequently observed following lymph node dissection in surgeries for gynecological malignancies. Functionally, the lymphatic system acts as a unidirectional drainage system, channeling 1.5-4 L of fluid back into the central circulation daily. The hepatic and enteral lymphatics generate approximately 80% of this fluid, with the remaining 20% produced by the upper and lower extremities [2]. A breakdown in this system can lead to a significant accumulation of lymph fluid in the abdominal cavity. If symptoms develop, they can severely debilitate patients, particularly those with a low performance status following the primary operation. The serious mechanical, nutritional, and immunological consequences of continuous protein and lymphocyte loss include pain, prolonged hospital stays (averaging 12.4-20.4 days), and an increase in the risk of infection, even in patients with negative microbiological tests [3]. Moreover, treatment of chylous ascites is often challenging and protracted. Conservative management is the initial approach, with lymphangiography and surgical ligation considered for refractory cases. However, no treatment currently guarantees a complete cure [4]. The pros of standby therapy are that invasive treatment can be avoided if the patient’s overall condition is relatively good. The cons are that after surgery for malignant tumors, when additional treatment such as chemotherapy is necessary, the start of treatment may be delayed due to lack of visibility. In this case, after two lymphangiograms, consent for invasive treatment was not obtained, resulting in standby therapy. We examined the usefulness of the standby therapy, which resulted in the improvement of refractory chylous ascites due to long-term intravascular retention of lymphangiograms.

## Case presentation

The patient was a 46-year-old woman with a history of two pregnancies and two live births. At the age of 42, she underwent surgery for the removal of colon cancer. Subsequently, due to a suspicion of left ovarian cancer, she underwent a simple total abdominal hysterectomy, bilateral salpingo-oophorectomy, partial omentectomy, pelvic lymph node dissection, and para-aortic lymph node dissection. During the surgery, she experienced leakage of clear, colorless lymphatic fluid from the lymph node dissection site. The lymph nodes were ligated using absorbable sutures (3-0 Polysorb®), which retain approximately 80% of their tensile strength for two weeks and 30% at three weeks post-implantation and are completely absorbed within 56-70 days. To conclude the procedure, a sheet-shaped biological tissue adhesive was applied to the para-aortic region, particularly around the lower border of the left renal vein. On the seventh postoperative day, ascites was noted in her pelvis during her discharge examination; however, she was asymptomatic and was discharged from the hospital. The final diagnosis was stage IA ovarian cancer and stage IA endometrial cancer, with the histopathological finding being grade 1 endometrioid carcinoma.

**Figure 1:**
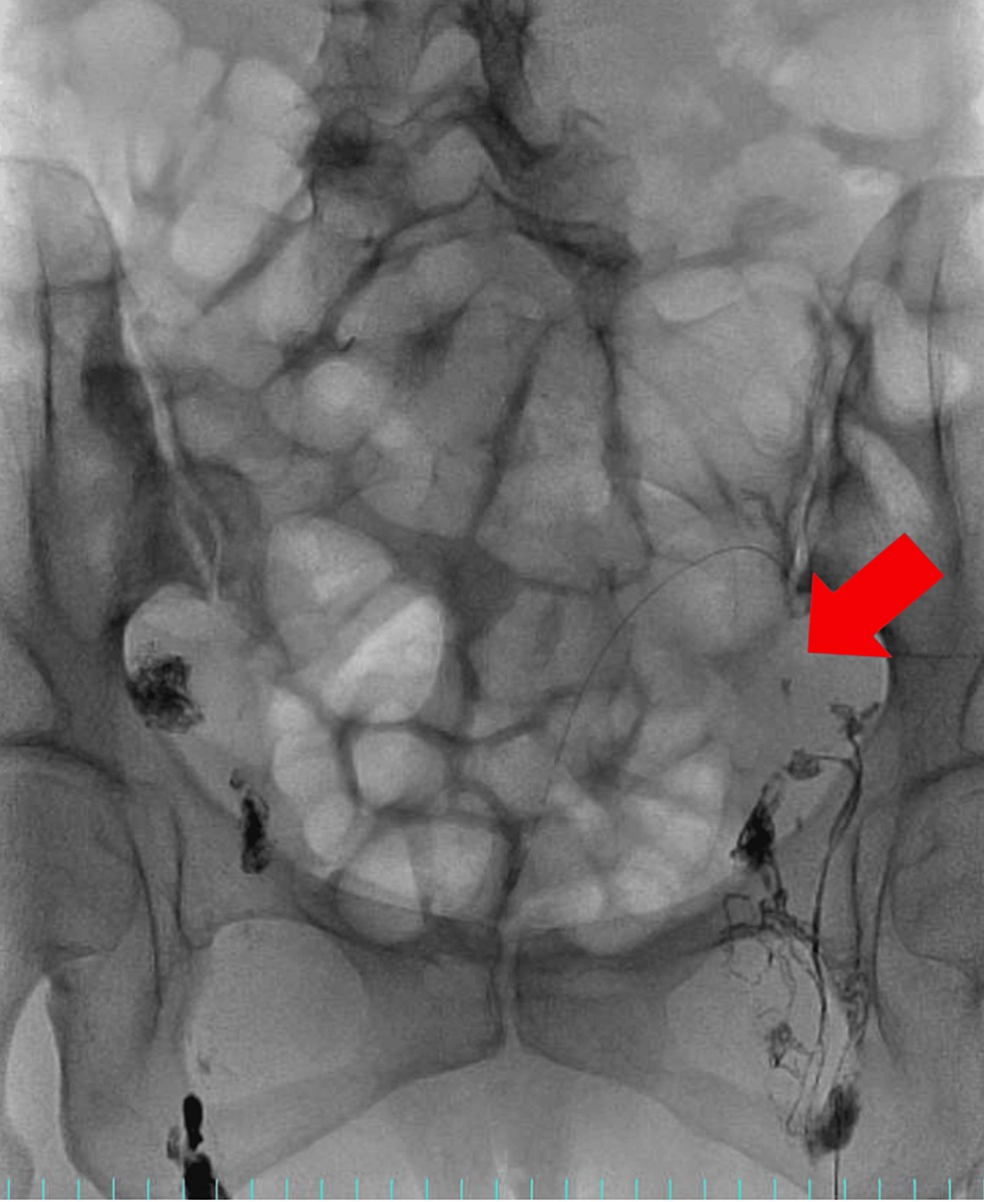
First lymphangiography. It was not possible to confirm that Lipiodol® reached the site of chyle ascites leakage, which had been previously identified by CT. The red arrow shows that the contrast agent could not reach the leak site.

**Figure 2:**
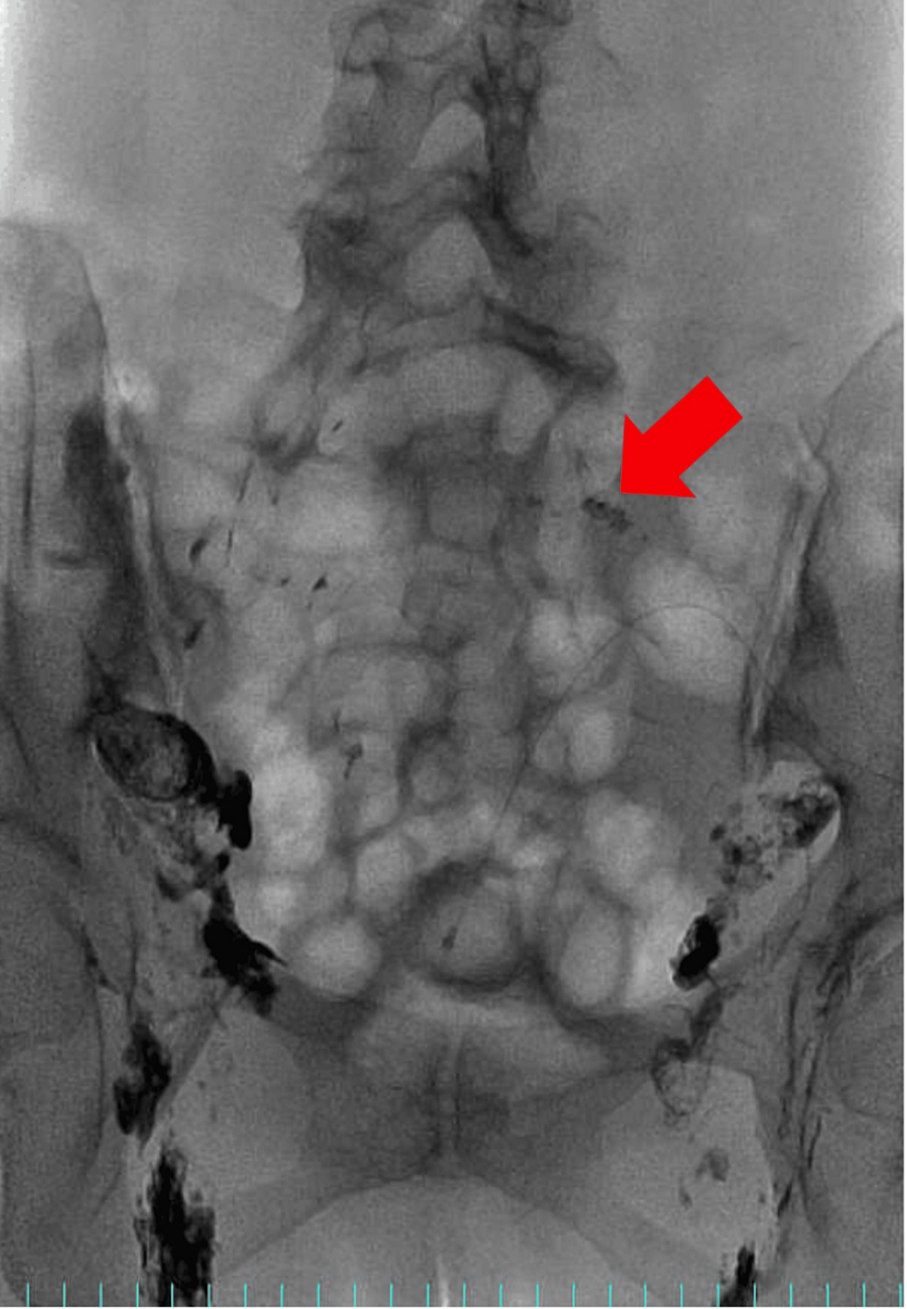
Second lymphangiography. Although stagnation was observed in some left lymph nodes, there was again no evidence of the fluid reaching the chyle ascites leakage site. The red arrow shows that the contrast agent could not reach the leak site again.

At the outpatient visit on the 31st postoperative day, transvaginal ultrasonic tomography confirmed significant amounts of ascites in the abdominal cavity. The patient reported significant abdominal distension and poor activities of daily living, leading to her emergency admission and the initiation of a fasting diet. Despite multiple ascites drainages facilitated by a drainage tube, there was no improvement. Therefore, on the 48th postoperative day, lymphangiography was performed. The procedure involved a percutaneous approach to bilateral inguinal lymph nodes using an ethyl ester of iodinated poppy-seed oil fatty acid (Lipiodol®) under fluoroscopic guidance. On the left side, 2 mL of Lipiodol® was used, revealing leakage at the left pelvic wall. However, the contrast agent did not reach the para-aortic lymph node, suspected to be the leakage site. On the right side, 4 mL was used with no observed leakage into the pelvis. Subsequent CT imaging showed stagnation in the lower abdominal wall lymph nodes without confirming that Lipiodol® reached the chyle. The patient was readmitted, and progress was monitored. With no evidence of improvement, a second lymphangiography was performed on the 54th postoperative day using 7 mL of Lipiodol®, and contrast imaging was done on seven lymph nodes in the inguinal region on both sides; however, none revealed the chylous leakage site (Figures 1, 2). There was also an indication that some lymphatic vessels had drained back into the veins, complicating further contrast imaging of the lymphatic vessels.

The patient’s symptoms improved the following day, but the chylous ascites persisted. She was recommended dietary therapy and continued hospitalization, but due to treatment intolerance, she was discharged at her request. During follow-up in the outpatient ward, her symptoms improved, and she was advised to restrict her fatty diet. However, a medical examination 114 days post-surgery revealed several chylous ascites. Interestingly, by the 142nd day post-surgery, the ascites had spontaneously resolved, and the patient was carefully monitored for 260 days post-surgery, with no recurrence of chylous ascites observed. As the chylous ascites disappeared, the persistently elevated cancer antigen 125 levels also decreased (Table 1).

**Table 1:** Chronological laboratory parameters. CEA = carcinoembryonic antigen; CA-19-9 = cancer antigen 19-9; CA-125 = cancer antigen 125

Laboratory parameter	Reference range	27th-day pre-surgery	31st-day post-surgery	48th-day post-surgery	142nd-day post-surgery
White blood cell (10^3^/µL)	4.00–8.60	7.28	3.31	4.82	3.97
Hemoglobin (g/dL)	12.0–16.0	12.5	13	12.8	11.2
Albumin (g/dL)	3.8–5.1	NA	3.1	2.8	NA
C-reactive protein (mg/dL)	<0.14	0.23	0.61	0.09	NA
CEA (ng/mL)	<5.0	1.3	NA	1.5	1.5
CA-19-9 (U/mL)	<37	30	NA	6	6
CA-125 (U/mL)	<35	540	NA	126	50

## Discussion

In this study, we explored several questions based on our case experience: can the onset of symptoms after gynecological surgical treatments be predicted based on certain risk factors? Do surgical techniques or medical equipment influence outcomes?

According to Zhao et al., the risk factors for chylous ascites within gynecology include pelvic lymph node dissection, with an incidence of 0.35% out of a total incidence of 0.9%, and para-aortic lymph node dissection, with an incidence of 4.08%. Given this frequency, whether para-aortic lymph node dissection was performed is considered significant [5]. Specifically, more than 14 para-aortic lymph node dissections were found to be a strong predictor of chylous ascites. Furthermore, chylous ascites resolved with conservative management in all documented patients [6].

Two patients who developed chylous ascites in our hospital in 2021 had undergone para-aortic lymph node dissection. Are there other factors that might trigger the onset of this condition? For lymph node dissection at our hospital, we performed en-bloc removal and ligated the coarse lymph nodes using 3-0 absorbable sutures. We also present a case involving a 59-year-old patient with endometrial cancer who underwent staging laparotomy at our hospital. Chylous ascites was noted one-week post-surgery; hence, fasting and subcutaneous injection of octreotide were initiated, yet no improvement was observed. However, lymphangiography on the 36th postoperative day showed an improvement in chylous ascites. As the tension retention and absorption periods of the absorbable sutures used in our surgeries were consistent, it is unlikely that the surgical operations or medical instruments had an impact. The pathological results in the other two cases were endometrioid carcinomas. Some reports suggest that underlying conditions that reduce effective circulating volume, such as liver cirrhosis and heart failure, may contribute to massive lymphatic ascites following retroperitoneal lymphadenectomy. Nonetheless, other reports in the gynecological field indicate no significant impact depending on the primary location or advanced stage of the malignant disease [7].

Factors contributing to the improvement in this case

In a conservative management study of 523 patients by Rose et al., 69% showed improvement in chylous ascites within a median of 11 days. However, they did not discuss the outcomes of unsuccessful cases. They also noted that lymphangiography and embolization led to an improvement rate of 85% [8]. Furthermore, a treatment algorithm for evidence-based conservative management has been presented, although it lacks mention of unsuccessful cases. Additionally, a review by Majdalany et al. indicated a high success rate for lymphangiography, with 18 out of 33 cases achieving leak visualization and 22 out of 33 cases achieving clinical success [9]. However, the subsequent treatment course for the clinically unsuccessful group remains undocumented.

In cases of chylous ascites, leakage lesions are often undetectable on lymphangiography because the intestinal lymph vessels that cause chylous leakage have a different flow path than those from the lower extremities [10].

The elimination half-life of the ethyl ester of iodinated poppy-seed oil fatty acid (Lipiodol®) used in lymphangiography is approximately 50 days. In this case, one of the factors contributing to symptom improvement might be that, even if lymphangiography shows no immediate effect, the agent remains in the lymph vessels for an extended period and may help block the leakage site.

There is also a case report where refractory chylous ascites improved using a small amount of an N-butyl 2-cyanoacrylate mixture without coils, suggesting this approach is worth considering [11]. Furthermore, in cases like this, where symptoms are relatively mild and surgical intervention is not preferred due to complications or patient preference following lymphangiography, it may be beneficial to use standby therapy in combination with dietary management during outpatient follow-up. Such an approach could yield medium- to long-term improvements and should be considered.

Is it possible to decide which treatment should be prioritized based on progress?

While various treatment options exist and there is no standard treatment protocol, the current mainstream approach begins with noninvasive treatments [3,5]. It is also widely accepted that treatment plans should be tailored to each patient, determined by the patient’s overall condition and symptoms. However, if further treatment is planned following the initial surgery, the patient’s long-term prognosis should be considered, and treatment should be administered promptly. Some reports have indicated that using a low-pressure drainage system results in improvement within nine days [12], making it a viable option for consideration when additional treatment is needed post-malignant surgery.

Limitations

The patient did not show improvement despite dietary therapy, percutaneous drainage therapy, and two lymphangiograms but exhibited improvement with elective therapy on the 142nd postoperative day. As this is a case report, its broader applicability and effectiveness are limited. Other methods, such as surgical lymphatic re-ligation, should be considered if additional treatment is required based on the clinical stage post-surgery or pathological results.

## Conclusions

Various methods exist for treating refractory chylous ascites, including expectant therapy, dietary management, percutaneous drainage, lymphangiography and embolization, and surgical lymphatic ligation. Tailoring individualized treatment plans for each patient and pursuing a multidisciplinary approach is advisable. Although initiating adjuvant chemotherapy may not be feasible, long-term standby therapy is beneficial, even if lymphangiography proves ineffective in the short term.
